# Revisited: *Borrelia burgdorferi* sensu lato infections in hard ticks (*Ixodes ricinus*) in the city of Hanover (Germany)

**DOI:** 10.1186/1756-3305-7-441

**Published:** 2014-09-18

**Authors:** Julia Tappe, Daniela Jordan, Elisabeth Janecek, Volker Fingerle, Christina Strube

**Affiliations:** Institute for Parasitology, University of Veterinary Medicine, Buenteweg 17, 30559 Hannover, Germany; German National Reference Centre for Borrelia, Veterinärstraße 2, 85764 Oberschleißheim, Germany

**Keywords:** *Borrelia burgdorferi* s.l, *Ixodes ricinus*, Vector-borne diseases, Tick-borne diseases

## Abstract

**Background:**

The present study investigated the prevalence of *Borrelia burgdorferi* sensu lato (s.l.) genospecies in *Ixodes ricinus* ticks collected in Hanover, Northern Germany, in 2010. At the same time the study served as fifth-year-follow-up study for data comparison with 2005.

**Methods:**

A total of 2100 questing ticks were collected and analysed by quantitative real-time PCR (qPCR) with subsequent species differentiation via Reverse Line Blot and Sanger sequencing. Simultaneously, results obtained in 2010 were compared to infection rates from 2005 to evaluate the development of *B. burgdorferi* s.l. infection rates in Hanoverian ticks.

**Results:**

Overall, 22.7% (476/2,100) of collected ticks were tested positive for *B. burgdorferi* s.l. infections. Adult ticks showed an infection rate of 33.3% (124/372), subdivided into 29.6% (58/196) positive males and 37.5% (66/176) positive females. Nymph and larvae infection rates were found to be 20.3% (344/1,697) and 25.8% (8/31), respectively. Species identification was successful for 59.2% (282/476) of positive ticks with *B. afzelii* as the most frequently detected genospecies, followed by *B. garinii* (including *B. bavariensis*) and *B. spielmanii. B. burgdorferi* sensu stricto (s.s.), *B. bissettii*, *B. valaisiana* and *B. lusitaniae* were also identified. Significant differences concerning seasonal fluctuations as well as local differences were observed. Comparing infection rates of Hanoverian ticks between years, a significant increase (P = 0.002) could be observed for larvae with 1.7% positives (2/60) in 2005 and 25.8% positives (8/31) in 2010. In the latter year, coinfections with *Borrelia* and Rickettsiales were detected in a total of 7.8% (163/2,100) of collected ticks. Of these, 7.3% (153/2,100) were coinfected with *Rickettsia* spp., 0.3% (7/2,100) with *A. phagocytophilum* and 0.1% (3/2,100) were coinfected with all three pathogens. Between years 2005 and 2010, no statistically significant differences in coinfection rates were found.

**Conclusions:**

Comparing *B. burgdorferi* s.l*.* infections in Hanoverian *I. ricinus* ticks in 2010 with data from 2005, a statistically significant increase of infected larvae was noted, whereas the other stages revealed no statistically significant differences. Whether the increased larvae infection rate is an isolated event or results from factual circumstances, e.g. increasing effectiveness of transovarial transmission due to unknown factors, has to be evaluated in further studies.

## Background

The hard tick *Ixodes ricinus* Linné 1758, known as vector for different pathogenic agents, serves as the main vector for spirochetes of the *Borrelia burgdorferi* sensu lato (s.l.) complex in Central Europe [1]. This genospecies complex includes the causative agents for Lyme borreliosis, the most frequent arthropod-borne human disease in the temperate northern hemisphere [2]. To date, 19 named spirochetes belong to the *B. burgdorferi* s.l. complex worldwide, however, several genospecies still remain unnamed [3–5]. In Europe, eleven genospecies of the *Borrelia burgdorferi* s.l. complex could be detected: *B. burgdorferi* sensu stricto (s.s.), *B. afzelii*, *B. bavariensis, B. bissettii*, *B. garinii*, *B. lusitaniae*, *B. spielmanii*, *B. valaisiana*, *B. kurtenbachii* (formerly included in the *B. bissettii* species), *B. finlandensis* and *B. carolinensis* [6–17]. All named species, with the exception of *B. finlandensis* and *B. carolinensis*, are implicated in different manifestations of Lyme borreliosis as, for example, erythema migrans, acrodermatitis chronica atrophicans, Lyme arthritis and neuroborreliosis [3, 5, 18].

In Europe, several reservoir hosts for Lyme disease-associated *Borrelia* spp. are known as, for example, small rodents, hedgehogs, squirrels, lizards and various bird species [16, 19–23]. Transmission of *B. burgdorferi* s.l. from ticks to hosts may occur through different developmental stages of *I. ricinus,* as transstadial transmission is very efficient. Even though larvae are occasionally affected, transovarial transmission in ticks seems to be inefficient [24]. Prevalence rates of *B. burgdorferi* s.l. in *I. ricinus* range from 6.1% in France to 22.5% in Western Switzerland [14, 25–28]. In Germany, prevalences of *Borrelia*-positive questing *I. ricinus* ticks range from 3.1% in Northern Germany to 27.0% in Thuringia or locally up to 37% in Bavaria [13, 29–33]. The present study was conducted to determine the *Borrelia* infection rate in Hanoverian ticks in 2010 and at the same time to serve as fifth-year-follow-up study to monitor changes or stagnation of tick infection rates when compared to data from 2005 [9]. For this, 2,100 ticks were collected in different recreational areas in 2010 in the northern German city of Hanover and subsequently analysed for *B. burgdorferi* s.l. infections.

## Methods

### Tick material

Questing ticks were collected each month from April to October 2010 in ten different recreation areas in the city of Hanover, the capital of the Northern German federal state Lower Saxony [34]. The city of Hanover was elected as the “German Capital of *Biodiversity*” in 2011 and is nicknamed “The green metropolis” as it houses numerous parks and the largest continuous urban woodland in Europe. At each sampling site, 30 ticks were collected per month resulting in 210 ticks per defined location over the sampling period and a total of 2,100 ticks overall. Tick species and developmental stages were determined microscopically based on morphological parameters [35].

### DNA isolation and detection of *Borrelia*spp

Genomic DNA isolation was carried out as described previously [34, 36]. Genomic DNA was eluted twice with 70 μl and 60 μl double-distilled water, respectively, to obtain a final volume of 100 μl genomic DNA. Detection of *B. burgdorferi* s.l. was achieved by minor groove binder probe-based qPCR targeting the 5S-23S intergenic spacer (IGS) region described by Strube *et al*. [37] with the modification that Absolute Blue QPCR low ROX mix (Thermo Fisher; containing Thermo-Start™ DNA Polymerase) was used and tick DNA template was increased to 10 μl. All qPCR reactions were performed as duplicates and each run included a negative template control as well as plasmid standard positive controls containing 10^0^ - 10^6^ copies of the *Borrelia* 5S-23S IGS and *Ixodes* ITS2 region, respectively [37]. Samples were resubjected to qPCR analysis, if only one well was determined positive and species identification was unsuccessful with the Reverse Line Blot (RLB). If no amplification could be detected in the second qPCR run, samples were considered as questionable positives.

### Identification of *B. burgdorferi*s.l. genospecies by Reverse Line Blot (RLB) and Sanger sequencing

Species identification of *Borrelia*-positive tick samples was achieved by RLB. Sanger sequencing was additionally applied to samples that were determined *B. garinii*-positive in RLB to further differentiate between *B. garinii* and *B. bavariensis*.

RLB was preceded by amplification of the *B. burgdorferi* s.l. 5S-23S IGS region using biotin linked forward primer 5SCB and reverse primer 23SN2 (10 pmol each) as described by Rijpkema *et al*. [38]. For the 25 μl reaction set up, 12.5 μl Thermo-Start™ PCR Master Mix (Thermo Scientific, Surrey, England), 1 μl of each primer and 5 μl tick DNA template were added to the corresponding amount of H_2_O. Cycling conditions were based on the protocol by Burri *et al*. [39] with addition of a polymerase activation step (15 min, 94°C). Each run included positive controls using template DNA of the following *Borrelia* genospecies (isolates): *B. afzelii* (PGau), *B. bavariensis* (PBi), *B. bissettii* (DN127), *B. burgdorferi* s.s. (Pka2), *B. garinii* (TN), *B. lusitaniae* (PotiB2), *B. spielmanii* (PHap) and *B. valaisiana* (VS116). Cross reactivity and specificity of the RLB were determined by using *B. duttonii*, *B. recurrentis* and *Treponema phagedenis* isolates as negative controls in addition to a non-template control.

RLB technique was performed as described by Rijpkema *et al*. [38] with few modifications: PCR products were hybridized to 7 different oligonucleotide probes: *B. afzelii* (AF; 10 μM), *B. garinii* (GA; 10 μM), *B. burgdorferi* s.s. (SS; 20 μM) [38], *B. bissettii* (BISNE2; 10 μM), *B. lusitaniae* (LUSINE2; 10 μM), *B. spielmanii* (SpiNE3; 800 μM) [40] and *B. valaisiana* (VSNE; 20 μM) [41]. Moreover, a modified probe for *B. burgdorferi* s.l. (SL2; 5’-[AmC6T]-CCATATTTTTATCTTCCATCTCTA-3’; 500 μM) was added as positive control for successful hybridization procedure. Furthermore, a probe for relapsing fever-like spirochetes (RFLNE; 250 μM) [40] was added to the RLB. All reactions were performed as duplicates.

Following sample hybridization as described by Rijpkema *et al*. [38], the membrane was washed with 2× SSPE-0.5% SDS at 45°C for 15 min, then incubated with streptavidin-peroxidase for 30 min at 42°C, and finally washed with 2× SSPE-0.5% SDS for 10 min at 42°C. Chemiluminescent hybridization signals were achieved by use of ECL detection reagent (GE Healthcare) and detected with Bio Imaging System MF-ChemiBIS 3.2 (Biostep, Jahnsdorf, Germany) during 2 to 10 min exposition (depending on signal strength). Reactions were performed as duplicates and all samples were tested at least twice.

**Figure 1:**
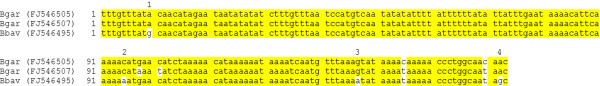
**SNPs in the partial 5S-23S intergenic spacer region of** ***B. garinii*** **and** ***B. bavariensis*** **.** SNPs used for genospecies differentiation are indicated by numbers. The displayed alignment is based on GenBank accession numbers FJ546505 (Bgar: *B. garinii*, strain IPT157), FJ546507 (Bgar: *B. garinii*, strain IPT165) and FJ546495 (Bbav: *B. bavariensis*, strain PBi).

To further differentiate between *B. garinii* and *B. bavariensis*, GA positive samples were reamplified and products showing visible gel bands were custom sequenced. Identification of those two species was based on four SNPs occurring in the 5S-23S IGS region of *B. bavariensis* and *B. garinii* (Figure 1).

Data was statistically analysed by application of chi-square test followed by Yates correction with SigmaStat® software (version 3.11) with subsequent Bonferroni-Holm correction. For analysis, α was defined with 0.05 and H_0_ was rejected if P ≤ 0.05. Ticks classified as questionable were included as positives in statistical analysis.

### Comparison of *B. burgdorferi*s.l. tick infection rates in 2010 vs. 2005

To compare results concerning infection rates of different tick stages obtained in 2010 to those obtained in 2005, questionable results were added to positive tested ticks in 2005 [9]. As a result, modified 2005 data was used for analysis as follows: 153 positive adults divided into 75 male and 78 female ticks (75 positive and 3 questionable), 96 nymphs (92 positive and 4 questionable) and 2 larvae (1 positive and 1 questionable). Data of adult and nymphal stages were statistically analysed as described above. Final significances were based on corrected α-values. To compare larval stages collected in 2005 with those collected in 2010, Fisher’s exact test was used due to low sample size. Final significances were based on corrected α-values.

### Coinfections of ticks with *B. burgdorferi*s.l. and Rickettsiales

To analyze the tick coinfection rates with *Borrelia* and Rickettsiales, obtained data on *B. burgdorferi* s.l. infections were compared with data on infections with Rickettsiales published previously [34]. Statistical analysis of present coinfections with *Rickettsia* spp. or *A. phagocytophilum* and comparison with data from 2005 was conducted as described above. Comparison of coinfections with all three pathogens between years was carried out by using Fisher’s exact Test (SigmaStat® software version 3.11).

## Results

Collection of 2,100 questing ticks resulted in 372 adult ticks (196 males and 176 females), 1,697 nymphs and 31 larvae all identified as *I. ricinus*. A total of 22.7% (476/2,100) of collected ticks was found to be infected with *B. burgdorferi* s.l.. Male adults showed an infection rate of 29.6% (58/196) whereas 37.5% (66/176) of female adults were determined positive resulting in an overall infection rate of 33.3% (124/372) for adult ticks. Nymphs showed an infection rate of 20.3% (344/1,697) and larval infection rates were determined as 25.8% (8/31). Statistically significant differences concerning developmental stages were observed between adults and nymphs (P < 0.001; α_loc_ = 0.017). On level below, adult males (P < 0.001; α_loc_ = 0.0083) as well as adult females (P = 0.003; α_loc_ = 0.01) were significantly more often infected than nymphs. A detailed overview of infection rates of different stages during the collection period is shown in Table 1.

**Table 1:** ***B. burgdorferi*** **s.l.-infected Hanoverian ticks (positive/total ticks) in 2010**

	April	May	June	July	August	September	October	Total
**Adults**	30/89	12/48	15/39	12/41	17/56	14/52	24/47	124/372
**(%)**	(33.7)	(25.0)	(38.5)	(36.6)	(29.3)	(26.9)	(51.1)	(33.3)
q*	1	0	2	0	1	0	1	5
**Thereof males**	15/57	6/29	8/19	7/24	7/24	6/25	9/18	58/196
**(%)**	(26.3)	(20.7)	(42.1)	(37.5)	(29.2)	(24.0)	(50.0)	(29.6)
q*	1	0	1	0	0	0	0	2
**females**	15/32	6/19	7/20	5/17	10/32	8/27	15/29	66/176
**(%)**	(46.8)	(31.6)	(35.0)	(35.6)	(31.3)	(29.6)	(51.7)	(37.5)
q*	0	0	1	0	1	0	1	3
**Nymphs**	27/201	41/248	61/261	47/259	46/244	52/241	70/243	344/1697
**(%)**	(13.4)	(16.5)	(23.4)	(26.6)	(18.9)	(21.6)	(28.8)	(20.3)
q*	4	5	4	2	2	4	4	25
**Larvae**	3/10	0/4	0/0	0/0	0/0	1/7	4/10	8/31
**(%)**	(30.0)	(0.0)	n.a.**	n.a.**	n.a.**	(14.3)	(40.0)	(25.8)
q*	3	0	n.a.**	n.a.**	n.a.**	0	2	5
**Total**	60/300	53/300	76/300	59/300	63/300	67/300	98/300	476/2100
**(%)**	(20.0)	(17.7)	(25.3)	(28.0)	(21.0)	(22.3)	(32.7)	(22.7)
q*	8	5	6	2	3	4	7	35

*q: questionable ticks. These ticks were included as positives in statistical analysis.

**n.a.: not applicable.

**Figure 2:**
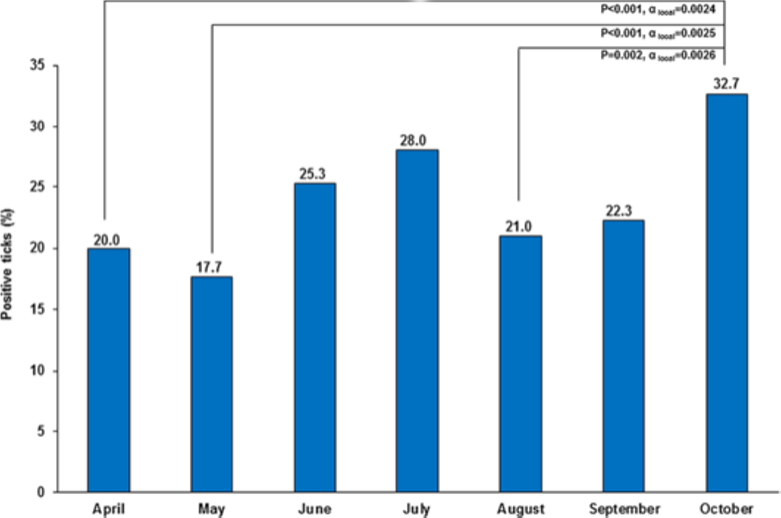
**Seasonal variations of** ***B. burgdorferi*** **s.l.-infected Hanoverian ticks in 2010.** Connection lines indicate significant differences between prevalence rates.

Over the collection period, two main infection peaks of ticks could be observed with a first increase in June (25.3%; 76/300) and July (28%; 83/300) as well as a second peak in October (32.7%; 98/300), the latter is simultaneously the highest seasonal prevalence. The lowest *Borrelia*-prevalence was determined in May (17.7%; 53/300). Statistically significant differences were observed between October and the months of April, May and August. P-values and adjusted α-values are shown in Figure 2.

**Figure 3:**
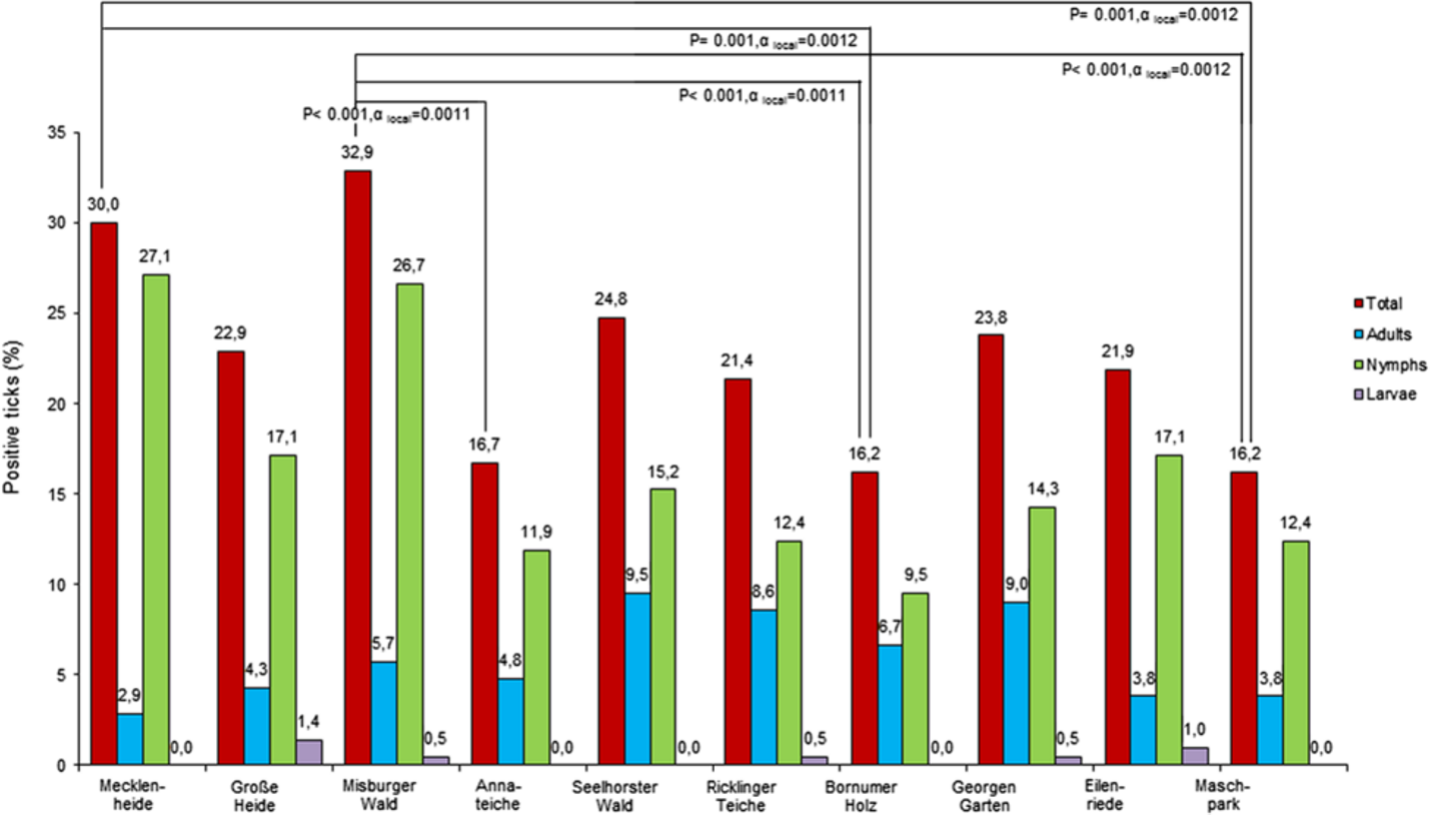
**Local distribution of** ***B. burgdorferi*** **s.l.-infected Hanoverian ticks in 2010.** Connection lines indicate significant differences between total prevalence rates.

**Figure 4:**
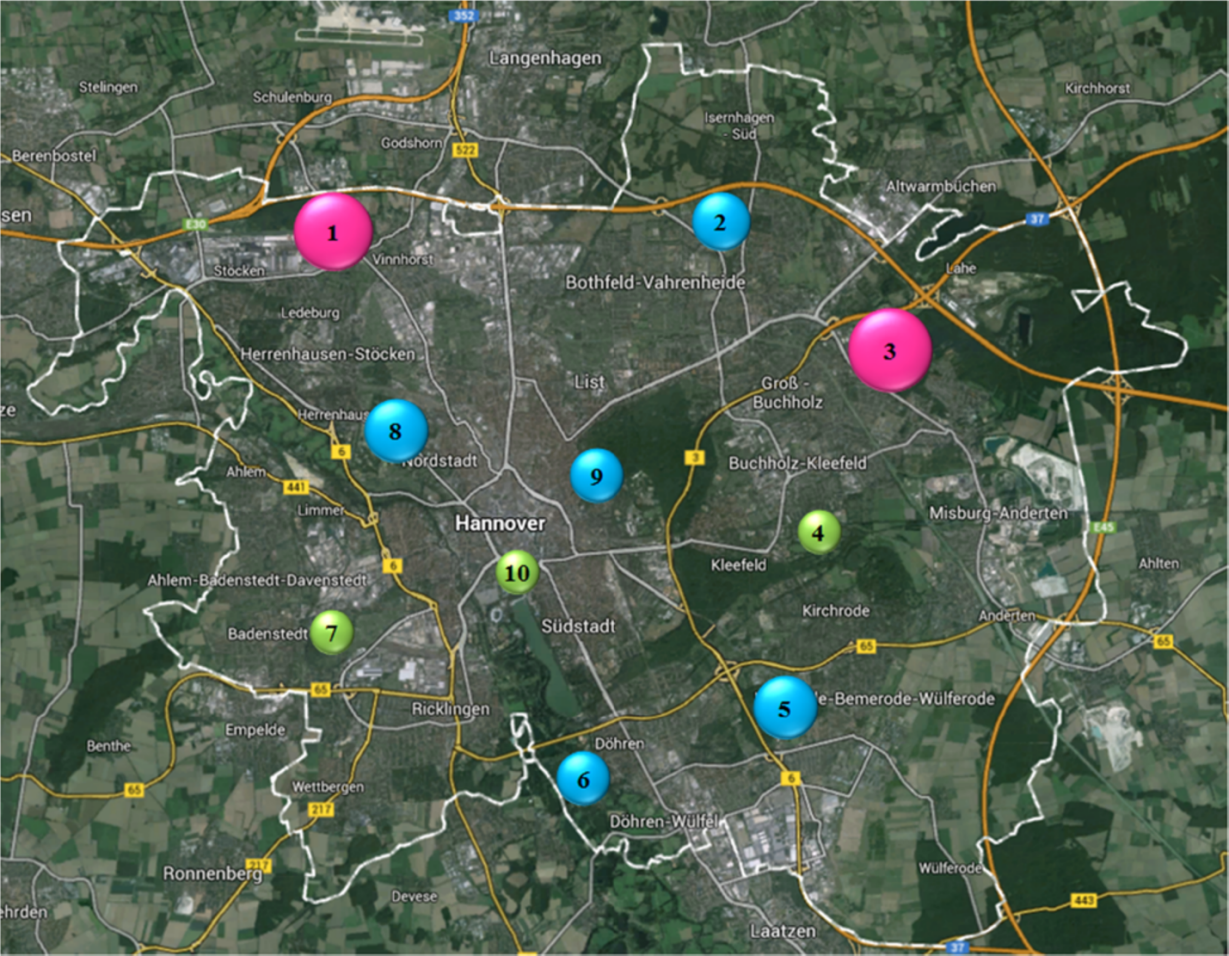
***B. burgdorferi*** **s.l.-infected Hanoverian ticks in 2010 at the different sampling locations.** Green dots: <20% infected ticks; blue dots: ≥20 < 30% infected ticks; pink dots: ≥30% infected ticks. 1: Mecklenheide; 2: Große Heide; 3: Misburger Wald; 4: Annateiche; 5: Seelhorster Wald; 6: Ricklinger Teiche; 7: Bornumer Holz; 8: Georgen Garten; 9: Eilenriede; 10: Maschpark. The white dashed line represents the city border (Map source: Google Earth).

Concerning the distribution of infected ticks at different sampling locations, several statistical significances were observed. The highest infection rate was found at “Misburger Wald” consisting of 32.9% (69/210) infected ticks, followed by “Mecklenheide” (30.0%; 63/210). The locations with lowest infection rates were “Bornumer Holz” and “Maschpark” (both 16.2%; 34/210) followed by “Annateiche” (16.7%; 35/210). Statistically significant differences were determined between “Misburger Wald” vs. “Bornumer Holz”, “Maschpark” and “Annateiche” as well as between “Mecklenheide” vs. “Bornumer Holz” and “Maschpark”. An overview of infection rates of tick stages at all sampling locations is provided in Table 2. Adjusted α-values and corresponding P-values are shown in Figure 3. *Borrelia* spp. infection rates in ticks at the different sampling locations are visualized in Figure 4.

**Table 2:** **Distribution of** ***B. burgdorferi*** **s.l.-infected ticks (positives/total ticks) at different collection sites in the city of Hanover in 2010**

	Mecklenheide	Große Heide	Misburger Wald	Annateiche	Seelhorster Wald	Ricklinger Teiche	Bornumer Holz	Georgen-garten	Eilenriede	Maschpark
**Adults**	6/21	9/22	12/35	10/27	20/42	18/46	14/44	19/53	8/36	8/46
**(%)**	(28.6)	(40.9)	(34.3)	(37.0)	(47.6)	(39.1)	(31.8)	(35.8)	(22.2)	(17.4)
q*	0	1	2	0	0	1	1	0	0	0
**Adult males**	2/12	2/6	7/17	4/16	7/19	11/28	5/21	10/27	6/26	4/24
**(%)**	(16.7)	(33.3)	(41.2)	(25.0)	(36.8)	(39.3)	(23.8)	(37.0)	(23.1)	(16.7)
q*	0	0	1	0	0	1	0	0	0	0
**Adult females**	4/9	7/16	5/18	6/11	13/23	7/18	9/23	9/26	2/10	4/22
**(%)**	(44.4)	(43.8)	(27.8)	(54.5)	(56.5)	(38.9)	(39.1)	(34.6)	(20.0)	(18.2)
q*	0	1	1	0	0	0	1	0	0	0
**Nymphs**	57/186	36/182	56/170	25/182	32/168	26/161	20/148	30/148	36/171	26/164
**(%)**	(30.6)	(19.8)	(32.9)	(13.7)	(19.0)	(16.1)	(13.5)	(20.3)	(21.1)	(15.9)
q*	4	2	3	2	3	3	3	2	1	2
**Larvae**	0/3	3/6	1/5	0/1	0/0	1/1	0/1	1/9	2/3	0/0
**(%)**	(0.0)	(50.0)	(20.0)	(0.0)	n.a.**	(100.0)	(0.0)	(11.1)	(66.7)	n.a.**
q*	0	1	1	0	0	1	0	0	2	0
**Total**	63/210	48/210	69/210	35/210	52/210	45/210	34/210	50/210	46/210	34/210
**(%)**	(30.0)	(22.9)	(32.9)	(16.7)	(24.8)	(21.4)	(16.2)	(23.8)	(21.9)	(16.2)
q*	4	4	6	2	3	5	4	2	3	2

*q: questionable ticks. These ticks were included as positives in statistical analysis.

**n.a.: not applicable.

### Identification and distribution of *B. burgdorferi*s.l. genospecies

In 65.5% (312/476) of *Borrelia* qPCR-positive ticks, RLB confirmed *B. burgdorferi* s.l. infection. Data was composed of 39 male adults, 40 female adults, 232 nymphs and 1 larva. Samples containing ≥10^4^ 5S-23S IGS copies showed a detection rate of 77.8% (7/9), ≥10^3^ copies 95.7% (44/46), ≥10^2^ 88.9% (96/108) and ≥10^1^ copies resulted in 74.2% (66/89) detection rate. Ticks containing ≤10 5S-23S IGS copies showed a detection rate of 44.2% (99/224). *B. burgdorferi* s.l. genospecies identification by RLB was successful in 282 of the 476 qPCR-positive ticks (59.2%). A detailed overview of detected genospecies is given in Table 3. Overall, *B. spielmanii, B. bissettii* and *B. lusitaniae* were mainly associated with multiple-infections.

**Table 3:** **Distribution of different** ***B. burgdorferi*** **s.l. genospecies in positive Hanoverian ticks in 2010**

Total infections	No. (% of infected ticks)	Mono-infections	No. (% of infected ticks)	Double-infection	No. (% of infected ticks)	Triple-/Quadruple-infection	No. (% of infected ticks)
Baf	147 (30.9%)	Baf	101 (21.2%)	Baf + Bbi	2 (0.4%)	Baf + Bga/Bba + Bsp	2 (0.4%)
Bva	46 (9.7%)	Bva	30 (6.3%)	Baf + Bga/Bba	11 (2.3%)	Baf + Bsp + Bss	1 (0.2%)
Bss	47 (9.9%)	Bss	25 (5.3%)	Baf + Blu	2 (0.4%)	Baf + Bss + Bva	1 (0.2%)
Bga/Bba	54 (11.3%)	Bga/Bba	29 (6.1%)	Baf + Bsp	12 (2.5%)	Bbi + Bga/Bba + Bsp	1 (0.2%)
Bsp	52 (10.9%)	Bsp	22 (4.6%)	Baf + Bss	10 (2.1%)	Bga/Bba + Bsp + Bss	1 (0.2%)
Blu	5 (1.1%)	Blu	2 (0.4%)	Baf + Bva	4 (0.8%)	Baf + Bga/Bba + Bsp + Bss	1 (0.2%)
Bbi	10 (2.1%)	Bbi	2 (0.4%)	Bga/Bba + Bbi	1 (0.2%)		
		No genospecies determined	194 (40.8%)	Bga/Bba + Bsp	3 (0.6%)		
				Bga/Bba + Bss	1 (0.2%)		
				Bga/Bba + Bva	4 (0.8%)		
				Bsp + Bbi	2 (0.4%)		
				Bsp + Blu	1 (0.2%)		
				Bsp + Bss	3 (0.6%)		
				Bsp + Bva	3 (0.6%)		
				Bss + Bbi	1 (0.2%)		
				Bss + Bva	3 (0.6%)		
				Bva + Bbi	1 (0.2%)		

Baf: *B. afzelii*, Bva: *B. valaisiana*, Bss: *B. burgdorferi* s.s., Bga/Bba: *B. garinii / bavariensis*, Bsp: *B. spielmanii*, Blu: *B. lusitaniae*; Bbi: *B. bissettii.*

Out of 54 tick samples with a positive signal using the *B. garinii* including *B. bavariensis* RLB probe, 44 samples were sequenced via Sanger sequencing and revealed 18.2% (8/44) *B. bavariensis*, 45.5% (20/44) *B. garinii* and 15.9% (7/44) non-identifiable samples. The remaining 20.5% (9/44) samples were assigned to other genospecies (*B. afzelii, B. burgdorferi* s.s*.*, *B. spielmanii* and *B. valaisiana*) corresponding to the genospecies coinfections identified by RLB.

### Coinfections of ticks with *B. burgdorferi*s.l. and Rickettsiales

The total coinfection rate of the 2100 analysed samples with Rickettsiales was 7.8% (153/2,100). Coinfections with *Rickettsia* spp. [*R. helvetica* in all typable samples (34)] were found in 7.3% (153/2,100) of the examined ticks and coinfections with *A. phagocytophilum* were detected in 0.3% (7/2,100) of ticks. Infection with all three pathogens was found in 0.1% (3/2100) of samples. Adult stages (43/372; 11.6%) were statistically significant (P = 0.005; α_loc_ = 0.01) more often infected with Rickettsiales than nymphs (120/1697; 7.1%). Concerning *Rickettsia* spp. coinfections, statistically significant differences (P = 0.002; α_loc_ = 0.01) were observed between adults (42/372; 11.3%) and nymphs (111/1,697; 6.5%). No coinfections were found in examined larvae. A detailed overview about infection rates and tick stages is provided in Table 4.

**Table 4:** **Coinfections with** ***B. burgdorferi*** **s.l. and Rickettsiales in Hanoverian ticks in 2010**

	No. of collected ticks	No. of ***B. burgdorferi***s.l. positive ticks	Total coinfections	***Rickettsia***spp. coinfections	***A. phagocytophilum***coinfections	Coinfections with ***Rickettsia***spp. and ***A. phagocytophilum***
		No. (%)	No. (%)	No. (%)	No. (%)	No. (%)
**Adults**	372	124 (33.3)	43 (11.6)	42 (11.3)	1 (0.3)	0 (n.a*.)
**Males**	196	58 (29.6)	22 (11.2)	22 (11.2)	0 (n.a*.)	0 (n.a*.)
**Females**	176	66 (37.5)	21 (11.9)	20 (11.4)	1 (0.6)	0 (n.a*.)
**Nymphs**	1697	344 (20.3)	120 (7.1)	111 (6.5)	6 (0.4)	3 (0.2)
**Larvae**	31	8 (25.8)	0 (n.a20F0.)	0 (n.a*.)	0 (n.a*.)	0 (n.a*.)
**All stages**	2100	476 (22.7)	163 (7.8)	153 (7.3)	7 (0.3)	3 (0.1)

*n.a.: not applicable.

### Comparison of tick infections with *B. burgdorferi*s.l. and coinfections with Rickettsiales between 2010 and 2005

Comparing infection rates of Hanoverian *I. ricinus* ticks with *Borrelia* spp. 5 years apart, the infection rate of adult stages and nymphs remained mainly unchanged with 35.5% (153/433) and 18.9% (96/507) infected individuals in 2005 [9] compared to 33.3% (124/372) and 20.3% (344/1,976) infected individuals in 2010. However, a statistically significant increase (P = 0.002) was observed for larval stages with 3.3% (2/60) infected larvae in 2005 whereas 25.8% (8/31) *Borrelia*-positive larvae were found in 2010. Concerning detection of different *B. burgdorferi* s.l. genospecies, it should be noted that in 2005, *B. garinii,* including *B. bavariensis,* was the most frequently detected genospecies, followed by *B. afzelii* and *B. spielmanii*. In 2010, *B. afzelii* was the most frequent detected species followed by *B. garinii* including *B. bavariensis* and *B. spielmanii*. Comparison of *Borrelia* spp. and Rickettsiales coinfection rates revealed no statistically significant difference between both years: Collected ticks were infected with *B. burgdorferi* s.l. and *Rickettsia* spp. at a rate of 9.1% (99/1,098) in 2005 [42] and 7.3% (153/2,100) in 2010. Coinfection rates with *B. burgdorferi* s.l. and *A. phagocytophilum* were 0.9% (15/1,646) in 2005 [43] and 0.3% (7/2,100) in 2010. Coinfections with all three pathogens were detected in 1.3% (5/391) of ticks in 2005 [42] and 0.1% (3/2,100) in 2010.

## Discussion

The hard tick *I. ricinus* serves as main vector for spirochetes of the *B. burgdorferi* s.l. complex in Central Europe. Parts of this complex are responsible for Lyme disease, a sickness that has increased rapidly during the past 20 years in the northern hemisphere [5, 44]. In several studies, infection rates of ticks with *B. burgdorferi* s.l. in Germany were investigated to assess the potential infection risk for humans resulting in a broad range of infection rates ranging from 3.1% in Northern Germany to 27.0% in Thuringia and 36.2% in Bavaria [29–33]. Besides data collection in different geographical regions, it is of importance to monitor tick infection rates over time to assess whether human infection risk increases or decreases. Thus, the present study served not only as a status survey for the Northern German state capital Hanover but also as fifth-year-follow-up survey of *Borrelia* infections in *I. ricinus* ticks.

The total infection rate of *B. burgdorferi* s.l.-infected ticks in 2010 was 22.7% with a significant difference between adults and nymphs (33.3% and 20.3% infected individuals, respectively), but not between adults and larvae (25.8% infected individuals). The expected highest burden in adult ticks is in accordance with different previous studies [6, 9, 31] and is most likely related to the combination of transstadial *Borrelia* transmission and the number of blood meals for development, which is connected to a higher probability to acquire bacteria from infected hosts. Comparison of *B. burgdorferi* s.l. infection rates in *I. ricinus* ticks of 2010 and 2005 resulted in approximately the same percentage of infected adults (33.3% in 2010 and 35.5% in 2005) and nymphs (20.3% in 2010 and 18.9% in 2005) whereas tick larvae showed a significantly different *B. burgdorferi* s.l. infection rate (P = 0.002) between 2005 (3.3%) and 2010 (25.8%). Due to low numbers of collected larvae (60 in 2005; 31 in 2010), the significant increase has to be verified in further studies and should be interpreted with caution. The rather high prevalence of *Borrelia*-infected larvae in 2010 might be explained by an interrupted blood meal with failed further development as well as by transovarial transmission. Even though transovarial transmission of *B. burgdorferi* s.l. was found to be rather inefficient [24, 45], it is the most plausible explanation as it seems unlikely that a quarter of collected larvae were removed during feeding from a *Borrelia*-infected host. However, solely in one of eight *Borrelia-*positive tested larvae, *B. burgdorferi* s.l. was confirmed by RLB. Overall, RLB confirmed 312 (65.5%) out of 476 *Borrelia* qPCR-positive ticks and identified 282 (59.2%) successfully concerning their genospecies. Reason for the generally lower RLB-positive rate is most likely a higher sensitivity of qPCR. The 30 tick samples in which RLB resulted in detection of *B. burgdorferi* s.l., but genospecies identification failed, might be explained by the higher sensitivity of the SL2 probe compared to genospecies-specific probes. Furthermore, not all in Europe occurring *B. burgdorferi* s.l. genospecies were detected by RLB – probes are not available for *B. kurtenbachii*, *B. carolinensis* and *B. finlandensis*. Moreover, ticks might have been infected with *Borrelia miyamotoi*, a *Borrelia* species associated with the relapsing fever group. *B. miyamotoi*, originally detected in Japan in *I. persulcatus* [46], was also previously found in *I. ricinus* ticks in Europe [47, 48]. In contrast to *B. burgdorferi* s.l., transovarial transmission could be demonstrated [49, 50]. However, the primer-probe-combination [9] used in the present study does not detect *B. miyamotoi*, but it is specific for the *B. burgdorferi* s.l. complex.

Regarding seasonal distribution of *B. burgdorferi* s.l. infection in Hanoverian *I. ricinus* ticks, the two peak course of infected ticks in June/July and October may depend on biological and climatic conditions like host disposability, temperature and humidity as these factors can affect pathogen transmission from hosts to ticks [51–54]. Concerning different sampling sites in the city of Hanover, tick infection rates range from 16.2% at the location “Maschpark” up to 32.9% at “Misburger Wald”. These differences in numbers of *Borrelia*-infected ticks might be explained by different incidences of *Borrelia*-infected hosts like small rodents or birds at single sampling locations.

*B. burgdorferi* s.l. genospecies identification resulted in findings comparable to results from previous studies, where *B. afzelii* was the predominant detected species in ticks [33, 55, 56], but also *B. garinii* including *B. bavariensis* and *B. spielmanii* were frequent findings in *Borrelia*-infected *I. ricinus* ticks [6, 12, 55]. Comparing mentioned genospecies distribution in 2010 with data from Hanoverian ticks collected in 2005, *B. afzelii*, *B. garinii* including *B. bavariensis* and *B. spielmanii* were the most frequently detected species in both studies. *B. afzelii* was the most mono-infecting species, *B. spielmanii* was found more often in multiple-infections than as mono-infecting species and frequent findings of double-infections contained a combination of *B. afzelii* and *B. spielmanii* [9]. Double- or multiple-infections (cf. Table 3) may result from blood meals on different hosts each infected with one genospecies, from one host having a multiple infection causing ticks to take up a number of genospecies, or from co-feeding of infected ticks. Several combinations including *B. afzelii* or *B. garinii* as detected in the present study were also observed by Pichon *et al.* [57]. The detected combination of *B. afzelii* and *B. burgdorferi* s.s. was also described amongst others in red squirrels [23].

Concerning coinfections of *Borrelia*-positive ticks with Rickettsiales in 2010, statistically significant differences were observed between different tick stages. This is in accordance with data from 2005, where adult ticks also showed higher infection rates than nymphs [42, 43]. No statistically significant differences in coinfection rates were found between years.

## Conclusion

In summary, comparing *B. burgdorferi* s.l. infections in Hanoverian *I. ricinus* ticks in 2010 with data from 2005, a significant increase of infected larvae was determined whereas nymphs and adult ticks did not show significantly changed infection rates. Whether this development of infected larvae is an actual fact through rather effective transovarial transmission under field conditions due to unknown factors, or an isolated event should be evaluated by further studies. The Hanoverian tick infection rate and distribution of *B. afzelii*, *B. garinii* and *B. spielmanii* as most abundant genospecies will be further monitored in the next fifth-year-follow-up study starting in April 2015.
