# Erratum to: Revisited: *Borrelia burgdorferi* sensu lato infections in hard ticks (*Ixodes ricinus*) in the city of Hanover (Germany)

**DOI:** 10.1186/s13071-016-1516-3

**Published:** 2016-04-29

**Authors:** Julia Tappe, Daniela Jordan, Elisabeth Janecek, Volker Fingerle, Christina Strube

**Affiliations:** Institute for Parasitology, University of Veterinary Medicine, Hanover, Buenteweg 17, Hannover, 30559 Germany; German National Reference Centre for Borrelia, Veterinärstraße 2, Oberschleißheim, 85764 Germany

## Erratum

Unfortunately, we noticed that in our published paper [1] the numbers of ticks co-infected with *Borrelia burgdorferi* (*sensu lato*) (*s.l*.) and *Anaplasma phagocytophilum* were partly incorrect as one adult female tick was detected false-positive for *A. phagocytophilum* infection. Corrected data showed that none of the female or adult ticks was co-infected with *Borrelia burgdorferi* (*s.l*.) and *Anaplasma phagocytophilum*. Among all tick stages, only six instead of seven out of 2,100 ticks were infected with both pathogens, but the prevalence of 0.3 % reported in [1] remained unchanged. The corrected Table 4 is included below. The authors would like to apologise for any inconvenience caused.

**Table 4:** Co-infections with *B. burgdorferi* (*s.l*.) and Rickettsiales in Hanoverian ticks in 2010

	No. of collected ticks	No. of *B. burgdorferi* (*s.l*.) positive ticks	Total co-infections	*Rickettsia* spp. co-infections	*A. phagocytophilum* co-infections	Co-infections with *Rickettsia* spp. and *A. phagocytophilum*
		No. (%)	No. (%)	No. (%)	No. (%)	No. (%)
Adults	372	124 (33.3)	43 (11.6)	42 (11.3)	0 (na*)	0 (na*)
Males	196	58 (29.6)	22 (11.2)	22 (11.2)	0 (na*)	0 (na*)
Females	176	66 (37.5)	21 (11.9)	20 (11.4)	0 (na*)	0 (na*)
Nymphs	1697	344 (20.3)	120 (7.1)	111 (6.5)	6 (0.4)	3 (0.2)
Larvae	31	8 (25.8)	0 (na*)	0 (na*)	0 (na*)	0 (na*)
All stages	2,100	476 (22.7)	163 (7.8)	153 (7.3)	6 (0.3)	3 (0.1)

*na, not applicable
